# Priapism as an Unusual Symptom of T-cell Acute Lymphoblastic Leukemia in a Pediatric Case

**DOI:** 10.7759/cureus.54331

**Published:** 2024-02-16

**Authors:** Mohammedalamin Mustafa, Ehab Hanafy, Shaima Riyad, Mustafa M Altoonisi, Waseem Aboulela

**Affiliations:** 1 Prince Sultan Oncology Center, King Salman Armed Forces Hospital, Tabuk, SAU; 2 Pediatrics Department, King Salman Armed Forces Hospital, Tabuk, SAU; 3 Urology Department, King Salman Armed Forces Hospital, Tabuk, SAU

**Keywords:** chemotherapy, hydroxyurea, leukapheresis, priapism, leukemia

## Abstract

Acute lymphoblastic leukemia (ALL) in pediatric patients typically presents with recognizable symptoms such as fever, pallor, and bone pain. However, atypical manifestations can complicate the diagnostic landscape. We present a unique case of a seven-year-old male with T-cell ALL whose presenting symptom was priapism. This case underscores the need for heightened awareness among healthcare professionals regarding the diverse clinical presentations of leukemia, emphasizing the importance of a multidisciplinary team approach for comprehensive evaluation and management.

Our seven-year-old patient presented with priapism. A comprehensive diagnostic workup, including complete blood counts and subsequent bone marrow examination, led to the diagnosis of T-cell ALL. Given the rare presentation, a multidisciplinary team consisting of pediatric oncologists/hematologists, urologists, and other relevant specialists collaborated to formulate a tailored treatment plan. The patient received an intensified chemotherapy regimen, resulting in the resolution of priapism and hematologic improvement.

Priapism as an initial presentation of T-cell ALL in a pediatric patient is an exceptional occurrence, necessitating a specialized and collaborative approach to diagnosis and management. This case report highlights the importance of interdisciplinary coordination involving pediatric oncologists and urologists in addressing the unique challenges posed by atypical leukemia presentations. The rarity of this manifestation emphasizes the need for further research to elucidate the underlying mechanisms and establish optimal management strategies for similar cases.

## Introduction

Priapism, a full or partial erection that continues for more than four hours beyond sexual stimulation and orgasm or is unrelated to sexual stimulation, is a rare but intriguing manifestation in the context of pediatric oncology [1]. The most common cause of priapism in children is sickle cell disease (65%), followed by leukemia (10%), trauma (10%), an idiopathic cause (10%), and a pharmacologically induced cause (5%). Its association with leukemia, particularly acute lymphoblastic leukemia (ALL), is a distinct and infrequently reported phenomenon [2]. Understanding the mechanism of priapism in the setting of leukemia and formulating an effective multidisciplinary approach to its management is paramount for providing optimal care to affected pediatric patients.

The pathophysiological link between priapism and leukemia remains incompletely understood. Leukemic infiltrates within the corpora cavernosa or altered blood rheology due to leukemia-induced hyperviscosity are proposed mechanisms contributing to priapism in these cases [3].

This case report focuses on a seven-year-old male presenting with priapism as the initial manifestation of T-cell ALL (T-ALL). Through an exploration of the underlying mechanisms and the collaborative efforts involved in the patient's management, we aim to shed light on the intricacies of this rare association. Emphasizing the importance of a multidisciplinary team comprising pediatric oncologists/hematologists and urologists, we elucidate the comprehensive approach required to navigate the complexities of priapism in the context of pediatric leukemia. This case not only highlights the unique challenges posed by atypical presentations of leukemia but also underscores the critical role of a cohesive and specialized healthcare team in ensuring optimal outcomes for the patients.

## Case presentation

A seven-year-old Saudi male child was admitted to our hospital presenting with neck swelling and a two-week history of fever, accompanied by abdominal pain, distension, fatigue, and reduced activity. Upon assessment, his oxygen saturation was recorded at 98% on room air, while his blood pressure was within the normal range for his age (110/75 mmHg), and he exhibited a heart rate of 80 beats per minute. Despite an analgesic intervention, the abdominal pain persisted for two days. The patient's parents were consanguineous, but there was no familial history of note. Notably, there were no prior instances of hypertension, diabetes, or other medically significant conditions, and the patient had not undergone any previous surgeries. Furthermore, there were no reported drug allergies. There was no history of any medical disorder that might initiate priapism as well. Developmentally, the child was on par with his age group, and his vaccinations were up to date.

Upon examination, there were bilateral enlarged non-tender cervical and axillary lymph nodes, and hepatosplenomegaly was evident, with both organs palpable 6 cm below the costal margin. A testicular examination revealed normal findings. Ear, nose, and throat examinations yielded unremarkable results, and the initial neurologic examination indicated normal motor power, sensations, and reflexes.

The initial laboratory results unveiled a markedly elevated total leukocytic count of 298 x10^3/µl, a hemoglobin level of 11.4 gm/dl, and a platelet count of 62x10^3/µl. A peripheral blood smear analysis indicated 82% blast cells, suggestive of acute leukemia.

Subsequently, the patient was admitted to the pediatric ICU (PICU) with a provisional diagnosis of acute leukemia, established based on clinical manifestations and initial laboratory findings. Further analysis indicated tumor lysis syndrome, with a significantly elevated uric acid level of 818 µmol/l (Table 1). Full supportive care, encompassing antibiotics, double maintenance intravenous fluids, and rasburicase, was initiated, resulting in the normalization of uric acid levels, while other electrolytes and renal function tests remained within acceptable ranges.

**Table 1:** Laboratory workup at admission and after two weeks

Laboratory parameter	Reference	Initial	Two weeks from admission
WBC	5.0-13.0	298 X 10^3/µl	1.9 X 10^3/µl
Hemoglobin	11.5-14	11.4 g/dl	10 g/dl
Platelets	180-400	62 X 10^3/µl	51 X 10^3/µl
Lactic acid dehydrogenase	141-237	2881 u/l	320 u/l
Sodium	136-145	138 mmol/l	133 mmol/l
Potassium	3.5-4.5	3.3 mmol/l	4.5 mmol/l
Creatinine	20-70	69 µmol/l	33 µmol/l
Uric acid	113-297	818 µmol/l	131 µmol/l
Aspartate aminotransferase	15-34	131 u/l	23 u/l
Alanine aminotransferase	12-49	52 u/l	48 u/l
Bilirubin	5-21	19 µmol/l	14 µmol/l

Throughout the hospital stay, comprehensive diagnostic procedures, including bone marrow aspiration (BMA), flow cytometry, and molecular studies, were conducted, confirming the diagnosis of T-ALL.

During admission, it was noticed that the patient had a sustained penile erection. An assessment revealed mild tenderness, and no discoloration was evident, indicative of painful priapism. The initial management approach encompassed hyperhydration, the commencement of hydroxyurea at a dosage of 20 mg per kg every six hours due to both high WBC count and priapism, a consultation with urology specialists, the initiation of dexamethasone at 10 mg per m2 per day, and an application of warm compressions. Despite these interventions, the priapism persisted. Considering the unavailability of leukapheresis, an attempted exchange transfusion was performed and proved unsuccessful. Subsequent urology recommendations for aspiration also failed to alleviate the condition.

Following the confirmation of the diagnosis through BMA and flow cytometry, and considering the normal results obtained from the initial lumbar puncture (LP) of the cerebrospinal fluid (CSF), the patient was started on chemotherapy. A gradual improvement in the priapism was noted during the induction phase of chemotherapy, with complete resolution achieved by day 13 of induction chemotherapy.

Meanwhile, the patient was risk stratified as ALL, T-cell, CNS-1, intermediate risk (because he received 2 doses of dexamethasone before diagnostic LP).

The follow-up at the end of the induction phase revealed negative minimal residual disease (MRD), and the patient is currently in good condition with no signs of priapism. Chemotherapy is being continued as per the Children's Oncology Group Trial AALL1231 protocol of chemotherapy for ALL.

## Discussion

The case of priapism as an unusual presentation of T-ALL in a pediatric patient unravels a multifaceted clinical scenario, demanding a nuanced exploration of diagnostic and therapeutic intricacies. Priapism, a rare phenomenon in the realm of pediatric oncology, adds a layer of complexity to an already intricate diagnostic landscape. The challenges posed by this atypical presentation, coupled with the limited success of conventional interventions, stimulate a comprehensive discourse on the optimal management strategies in such cases.

Hydroxyurea, a myelosuppressive agent with established efficacy in addressing priapism related to sickle cell disease and certain malignancies [4], failed to provide immediate relief in our case of leukemia-induced priapism. This underscores the enigmatic nature of priapism associated with T-ALL, highlighting the need for a tailored therapeutic approach beyond conventional measures. Similarly, corticosteroids play a crucial role in the treatment of priapism, especially in the context of leukemia. In cases where priapism is associated with leukemia, dexamethasone is often incorporated into the therapeutic approach due to its anti-inflammatory and vasoconstrictive properties. The steroid's ability to reduce inflammation and modulate immune responses contributes to its effectiveness in managing the underlying causes of priapism, particularly when leukemia-related complications are involved. Dexamethasone's inclusion in the treatment regimen, alongside other interventions such as hydration and urologic consultations, aims to alleviate symptoms and prevent the persistence of priapism. This multifaceted approach, guided by the specific considerations of leukemia-associated priapism, underscores the importance of a comprehensive and tailored medical strategy for optimal patient outcomes. [5].

Urologic interventions, including aspiration and irrigation, represent standard approaches to relieve ischemia in priapism [6]. The lack of an immediate response to these interventions in our case underscores the complexity of priapism associated with T-ALL. The refractory nature of the priapism observed might be attributed to leukemic infiltration compromising vascular integrity, emphasizing the imperative for targeted hematologic interventions.

The noteworthy aspect of our case lies in the gradual resolution of priapism following the initiation of chemotherapy. The potential mechanisms behind this resolution involve the reduction of leukemic burden and the consequent alleviation of vascular compromise. The intricate interplay between leukemic infiltration within the corpora cavernosa and hyperviscosity-induced changes in blood rheology may contribute to the persistent nature of priapism [7,8]. The cytoreductive effects of chemotherapy, a cornerstone in leukemia management, emerge as a pivotal factor in restoring normal erectile function.

Comparisons with existing literature reveal a lack of consensus on the optimal management of leukemia-induced priapism. Case reports documenting successful resolution with chemotherapy, such as imatinib in chronic myeloid leukemia [9], underscore the importance of tailoring interventions based on the specific leukemic subtype. Our case, requiring a multidisciplinary approach involving oncologists/hematologists and urologists, reflects the evolving understanding of leukemia-induced priapism and emphasizes the need for collaborative efforts to optimize patient care.

Leukapheresis, an extracorporeal therapy involving the removal of WBCs from the bloodstream, emerges as a potential adjunctive measure in cases of priapism associated with elevated WBC counts. This therapeutic approach aims to rapidly reduce the leukemic burden and mitigate microvascular occlusion contributing to priapism. Although leukapheresis has demonstrated success in managing complications of hyperleukocytosis in acute leukemia, its specific role in the context of priapism remains to be precisely defined [10]. Further research is warranted to elucidate the potential benefits and optimal timing of leukapheresis in leukemia-induced priapism.

In conclusion, our case report contributes to the evolving understanding of the complexities surrounding priapism as an initial presentation of T-ALL in pediatric patients. The lack of immediate response to traditional interventions and the subsequent improvement with chemotherapy highlight the unique challenges posed by leukemia-associated priapism. This extensive discussion emphasizes the need for tailored, multidisciplinary approaches to optimize patient outcomes and underscores the importance of ongoing research to refine and standardize the management protocols for this rare and complex manifestation.

## Conclusions

The presented case of priapism as an initial manifestation of T-ALL in a pediatric patient underscores the rarity and diagnostic challenges associated with this atypical presentation. Conventional interventions, including hydroxyurea and urologic procedures, demonstrated limited efficacy, emphasizing the intricate nature of leukemia-induced priapism. The gradual resolution observed upon the initiation of chemotherapy highlights the pivotal role of hematologic management in achieving successful outcomes. This case reinforces the need for a multidisciplinary approach, involving oncologists and urologists, to navigate the complexities of leukemia-associated priapism. Further research is warranted to refine management protocols and elucidate the precise role of interventions such as leukapheresis in optimizing outcomes for these rare cases.
